# A dataset of criteria on the use of thermal insulation solutions in building facades located in Norway, Portugal and Italy

**DOI:** 10.1016/j.dib.2023.109622

**Published:** 2023-09-26

**Authors:** J.L. Parracha, B. Bartolucci, G. Boccacci, O. Ogut, G. Bartels, A.M. Siani, F. Frasca, C. Bertolin, M.P. Mendes, I. Flores-Colen

**Affiliations:** a CERIS, Department of Civil Engineering, Architecture and Environment, Instituto Superior Técnico, University of Lisbon, Lisbon, Portugal; b LNEC, Department of Buildings, National Laboratory for Civil Engineering, Lisbon, Portugal; c Department of Earth Sciences, Sapienza University of Rome, Rome, Italy; d Department of Mechanical and Industrial Engineering, Norwegian University of Science and Technology, Trondheim, Norway; e Department of Architecture, Built Environment and Construction Engineering, Politecnico di Milano, Milan, Italy; f Department of Physics, Sapienza University of Rome, Rome, Italy; g CIABC Research Center, Sapienza University of Rome, Rome, Italy; h Department of Civil Engineering, Architecture and Environment, Instituto Superior Técnico, University of Lisbon, Lisbon, Portugal

**Keywords:** Energy efficiency, Thermal comfort, Questionnaire survey, Thermal insulation materials, PESTE criteria, Performance parameters

## Abstract

The building sector is responsible for a significant percentage of the energy consumption in Europe. The level of thermal insulation of the building envelope leads to decrease energy consumption, thus contributing towards a sustainable and efficient built environment. As a result, the choice of the most suitable thermal insulation solution to be applied both in new construction and in retrofitting of building facades is fundamental for a satisfactory thermal performance of the building. Nevertheless, the thermal insulation solution should not be chosen considering only the thermal performance, but rather based on a set of performance parameters (i.e., water resistance, fire performance, impact on the environment and human health, among others) and climate-related requirements. This data article includes a dataset on criteria adopted in three European countries (namely Norway, Portugal, and Italy) considering a PESTE analysis (i.e., criteria related to Political, Economic, Social, Technological, and Environmental questions). The main objective was to evaluate the knowledge and perception of people living and/or working in these countries about the use and the performance of thermal insulation solutions in building facades. To this aim a questionnaire was developed within the scope of the EEA Granted EFFICACY research project (November 2022 – February 2023), whose overall objective is to create a database that serves as a reference for the choice of thermal insulation solutions to be applied in building facades for thermal and energy performances optimization. This database contributes to systemize criteria and can be extended by other researchers or professionals in the area, as well as in other countries.

Specifications Table

**Table:** 

Subject	Civil Engineering; Building Engineering
Specific subject area	Building energy performance; Thermal retrofitting of facades; Thermal insulation solutions
Data format	Raw
Type of data	Excel spreadsheet
Data collection	Data were collected responding to a questionnaire survey developed within the scope of the EEA Granted EFFICACY (Energy eFFiciency building and CirculAr eConomY for thermal insulating solutions) project and considering a PESTE analysis (i.e., the questionnaire was designed considering 5 different sections related to **P**olitical, **E**conomic, **S**ocial, **T**echnological, and **E**nvironmental questions). Demographic and calibration information (e.g., age, gender, nationality, level of education, among others) was also considered. The questionnaire survey was delivered online, mainly in three European countries (i.e., Norway, Portugal, and Italy) in the period between November 2022 and February 2023. The questionnaire was edited in English, Italian and Portuguese. The questionnaire asked people living and/or working in each country to express their perception and knowledge about the use and the performance of thermal insulation solutions applied in new construction and in thermal retrofitting of building facades. The respondents were clustered considering their level of specialization and knowledge about thermal insulation solutions.
Data source location	Mainly in three European countries (Norway, Portugal, and Italy) but also in other countries (e.g., Brazil).
Data accessibility	Raw data were deposited at Mendeley dataset website and are available at the following link: https://data.mendeley.com/datasets/z8sphs8vvv/2

## 1 Value of the Data

• The data provide a new understanding on the use of thermal insulation solutions in building facades located in Norway, Portugal and Italy considering the knowledge and perception of people living and/or working in those three European countries.
• The data provide both qualitative and quantitative information on the level of knowledge of the respondents related with thermal insulation performance criteria, as well as the needs regarding thermal comfort inside their dwellings.
• The data can be useful for researchers working in the field of the energy efficiency of buildings and thermal insulation materials who seek to further understand the use of different thermal insulation solutions in new construction and in thermal retrofitting of Norwegian, Portuguese and Italian facades considering stakeholders knowledge and perception. The data is also valuable for building professionals, manufacturers of thermal insulation solutions, and others interested in energy efficiency and building thermal retrofitting.

## 2 Data Description

**Fig. 1:**
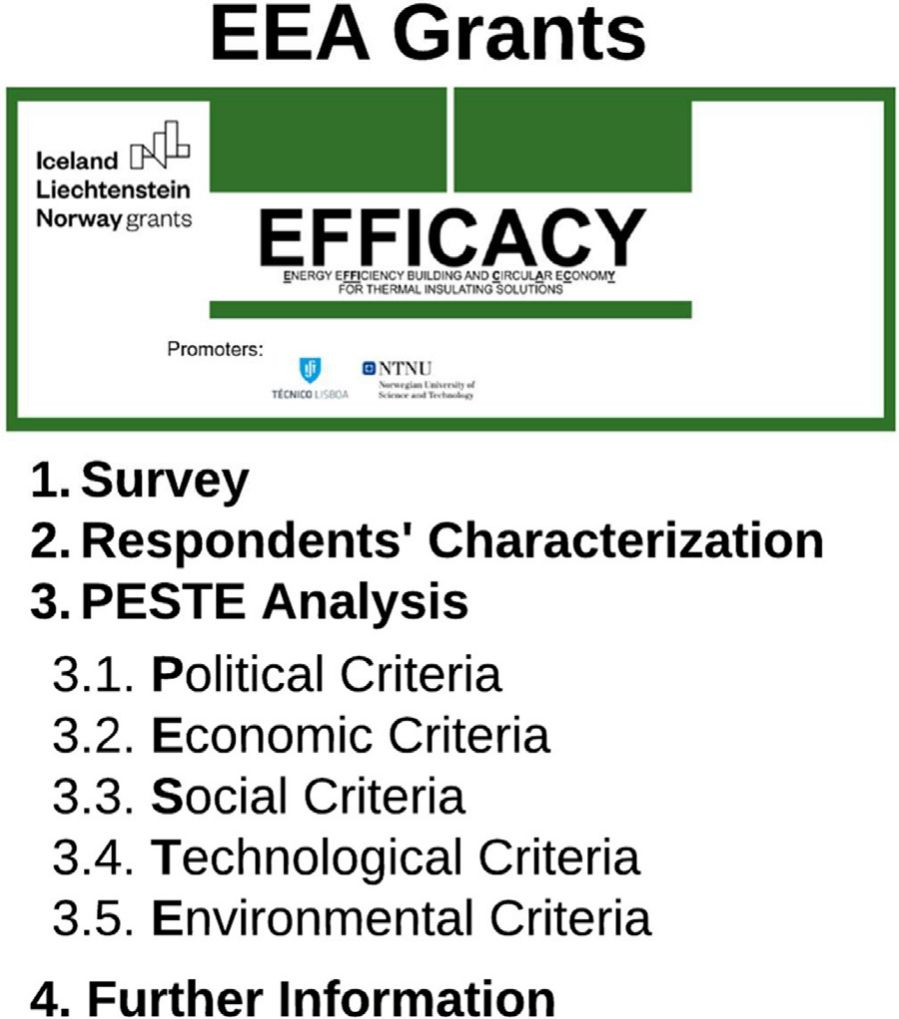
Main contents of EFFICACY project dataset.

The dataset consists of an Excel spreadsheet divided into ten sheets, i.e., an introductory sheet and nine sections and sub-section, as reported in Fig. 1. The questionnaire was developed based on the PESTEL analysis which is a widely used framework in business and strategic planning in the construction sector [1,2]. It assesses external factors that influence people's perception of thermal insulation solutions in building facades across different countries. The questionnaire survey was prepared considering five sections out of six of the complete PESTEL analysis, hereafter called as PESTE **(P**olitical, **E**conomic, **S**ocial, **T**echnological, **E**nvironmental) after the removal of the “L” component corresponding to the **L**egislative section (i.e., rules and regulations) not considered within the survey. In the scope of this study, it was considered that questions related to Legislative criteria are strongly associated with Political criteria. Therefore, the question related with legislation (i.e., “Are you aware of any national/international directive on energy retrofitting; please name the directive”) was included in the Political section. The online questionnaire included a total of 67 questions divided into five sections (Fig. 1).

In section “2. Respondents’ characterization”, information about the respondents was collected with the objective of characterizing the sample. In this sheet, the respondents were characterized considering their work category, age, gender, nationality, living country, country's region, job sector, job experience, country of professional activity, level of education, living place population and landform. There were also items related to the respondents building ownership type (e.g., if owner or tenant), their type of house and characteristics, and information about the energy retrofitting interventions occurrence in their building if priorly done or planned in the future.

In section “3. PESTE analysis”, political, economic, social, technological, and environmental questions were addressed. In the Political section, e.g., respondents were asked if they are aware of government financial incentives for retrofitting interventions, and if they think those incentives are adequate. Then, some Economic questions were asked, where respondents demonstrated their willingness to retrofit their houses according to some factors such as possible incentives from the state and payback period. Afterwards, some Social questions related with thermal comfort and the presence of mould or humidity inside the building were addressed. The respondents, with intermediate (between 5 and 10 years) or expertise (more than 10 years) knowledge on thermal insulation materials, were also questioned about Technological aspects, regarding selection criteria of different thermal insulation materials considering several performance parameters (i.e., durability, fire behavior, needs of maintenance, market price, biological colonization, water and mechanical performances, and sustainability). A questionnaire-based rating system was developed in this section, with people rating several of the most used thermal insulation solutions considering their performance characteristics (Table 1). Finally, the Environmental questions addressed aspects such as climate change and the environmental impact of the different phases of the life cycle of thermal insulation solutions.

**Table 1:** Questionnaire-based rating system to rate the performance of the most used thermal insulation solutions.

Thermal insulation solutions	Performance parameters	Questionnaire-based rating system (from 1 to 5)
Insulation cork board (ICB) Mineral wool (MW) Expanded polystyrene (EPS) Extruded polystyrene (XPS) Polyurethane foam (PUR) Natural fibers (NF) Aerogel blankets (AB) Thermal insulating mortars (TM) Vacuum-insulation panels (VIP) Vegetation – green walls (VEG)	Durability	1 – less durable; 5 – most durable
	Market price	1 – less expensive; 5 – most expensive
	Needs of maintenance	1 – lowest need; 5 – highest need
	Fire behavior	1 – worst performance; 5 – best performance
	Biological colonization	1 – lowest bio-susceptibility; 5 – highest bio-susceptibility
	Water performance	1 – lowest water retention; 5 – highest water retention
	Mechanical performance	1 – lowest mechanical resistance; 5 – highest mechanical resistance
	Sustainability	1 – most sustainable; 5 – least sustainable

## 3 Experimental Design, Materials and Methods

The building sector is responsible for more than 40% of the energy consumption in Europe, with 27% attributed to the residential sector [3]. To deal with this problem, several EU directives were issued with the aim at decreasing the energy demand of existing buildings and improving the energy performance of new buildings. A previous study [4] estimates that more than 90% of existing dwellings will still exist in 2050, which is the year defined by the EU for the achievement of climate-neutral building stock.

Thermal insulation solutions have already been proved as one of the best strategies for reducing energy consumption and losses in buildings [5]. Therefore, the choice of the most suitable thermal insulation solution to be applied is of fundamental importance for an adequate thermal performance of the building. However, the thermal insulation solution should not be chosen considering only the thermal performance, but rather based on a set of performance parameters (i.e., water resistance, fire performance, impact on the environment and human health, among others) and climate-related requirements for each country with different application conditions.

**Fig. 2:**
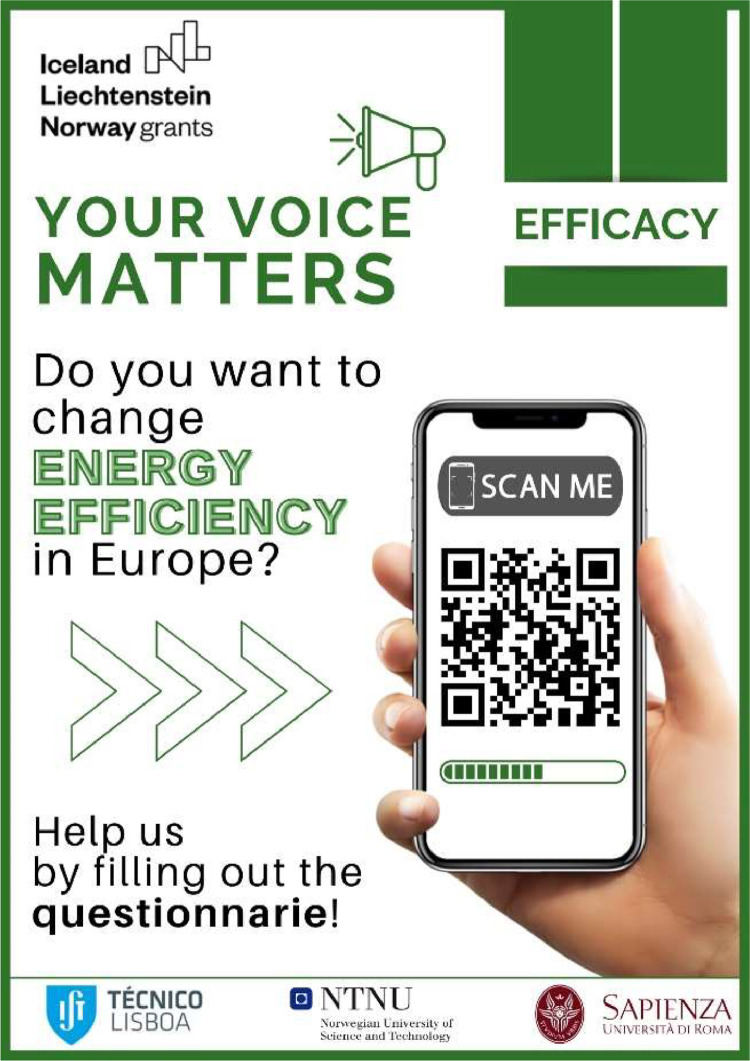
Poster displayed at universities in Norway, Portugal and Italy.

This study gathered data from an online questionnaire survey on the use of thermal insulation solutions for new construction and thermal retrofitting of building facades mainly in Norway, Portugal, and Italy. The questionnaire was prepared using Google Forms and was edited in English, Italian and Portuguese. The questionnaire was disseminated online via email and social media (LinkedIn, Twitter, Facebook, and Instagram) in the period between November 2022 and February 2023. A poster with the questionnaire survey available via a QR code (Fig. 2) was also displayed at the authors’ universities (i.e., Norwegian University of Science and Technology in Norway; Instituto Superior Técnico in Portugal; and Sapienza University of Rome in Italy). In order to avoid duplicate responses, the questionnaires included a section for participants to enter their email addresses. Additionally, all responses were initially screened to identify possible duplicates, which were removed from the sample. This condition was not subject to any further control.

The main objective of the study was to analyze the level of knowledge and perception of the respondents related with thermal insulation performance, as well as the needs regarding thermal comfort inside their dwellings. The questionnaire was developed by the research team of the EEA Granted EFFICACY (Energy eFFiciency building and CirculAr eConomY for thermal insulating solutions) project considering the relationships among the various **P**olitical, **E**conomic, **S**ocial, **T**echnological, and **E**nvironmental factors (i.e., PESTE analysis) involved in the choice of thermal insulation solutions. Demographic and calibration information (e.g., age, gender, nationality, level of education, among others) was also considered. The respondents were randomly selected to have a wider range of answers from professional and non-professional figures. Nevertheless, particular focus was put on stakeholders, researchers working in the field of the energy efficiency of buildings and thermal insulation materials, private research institutions and non-profit associations working on energy efficiency of buildings and circularity, building professionals, manufacturers of thermal insulation solutions and university professors and students from related fields (e.g., energy efficiency, circular economy, social impacts, climate change).

A total of 221 respondents completed the entire questionnaire and the replies were all included in the database; 203 responses were related to people living and/or working in Norway, Portugal, or Italy (i.e., in detail 24 from Norway, 127 from Portugal and 52 from Italy,). As it can be observed, the number of responses obtained in Portugal and Italy is significantly higher when compared to Norway. This is partially because of the dissemination channels of the questionnaire survey, which are mainly connected to the university. The Norwegian University of Science and Technology has a high number of foreign students who do not live permanently in Norway, thus lowering the number of responses. Therefore, in order to increase the Norwegian sample, other dissemination channels should be considered in the future. However, when considering only the 24 responses from Norway, certain expected trends have already emerged. For example, 77% of the Norwegian respondents have thermal insulation in their buildings, a percentage significantly lower for the Portuguese (52%) and Italian (37%) respondents, which is in line with previous survey/study findings [6].

Raw data were deposited at Mendeley dataset and can be found online at: https://data.mendeley.com/datasets/z8sphs8vvv/2.

## Limitations

The number of responses obtained in Portugal and Italy is significantly higher when compared to Norway. Therefore, the Norwegian sample should be increased in future research.

## Ethics Statement

All respondents were protected by providing full transparency on the motivation and purpose of the research and ensuring the anonymity of participants following the General Data Protection Regulation (GDPR) – Regulation (EU) 2016/679 directive [7]. No further ethics statements are applicable in the present study.

## CRediT authorship contribution statement

**J.L. Parracha:** Conceptualization, Methodology, Validation, Formal analysis, Data curation, Writing – original draft, Visualization. **B. Bartolucci:** Conceptualization, Methodology, Validation, Data curation, Writing – review & editing. **G. Boccacci:** Methodology, Writing – review & editing. **O. Ogut:** Conceptualization, Methodology, Validation, Data curation, Writing – review & editing. **G. Bartels:** Data curation, Visualization. **A.M. Siani:** Conceptualization, Methodology, Writing – review & editing. **F. Frasca:** Conceptualization, Methodology, Writing – review & editing. **C. Bertolin:** Conceptualization, Methodology, Writing – review & editing, Funding acquisition. **M.P. Mendes:** Conceptualization, Methodology, Validation, Writing – review & editing. **I. Flores-Colen:** Conceptualization, Methodology, Validation, Writing – review & editing, Funding acquisition, Project administration.

## Data Availability

The EFFICACY Project database (Original data) (Mendeley Data)
